# A Characterization of the Electrophysiological and Morphological Properties of Vasoactive Intestinal Peptide (VIP) Interneurons in the Medial Entorhinal Cortex (MEC)

**DOI:** 10.3389/fncir.2021.653116

**Published:** 2021-07-23

**Authors:** Saishree Badrinarayanan, Frédéric Manseau, Sylvain Williams, Mark P. Brandon

**Affiliations:** ^1^Department of Psychiatry, Douglas Hospital Research Centre, McGill University, Montreal, QC, Canada; ^2^Integrated Program in Neuroscience, McGill University, Montreal, QC, Canada

**Keywords:** VIP interneurons, medial entorhinal cortex, *in vitro* patch clamp, characterization, interneurons

## Abstract

Circuit interactions within the medial entorhinal cortex (MEC) translate movement into a coherent code for spatial location. Entorhinal principal cells are subject to strong lateral inhibition, suggesting that a disinhibitory mechanism may drive their activation. Cortical Vasoactive Intestinal Peptide (VIP) expressing inhibitory neurons are known to contact other interneurons and excitatory cells and are thus capable of providing a local disinhibitory mechanism, yet little is known about this cell type in the MEC. To investigate the electrophysiological and morphological properties of VIP cells in the MEC, we use *in vitro* whole-cell patch-clamp recordings in VIPcre/tdTom mice. We report several gradients in electrophysiological properties of VIP cells that differ across laminae and along the dorsal-ventral MEC axis. We additionally show that VIP cells have distinct morphological features across laminae. Together, these results characterize the cellular and morphological properties of VIP cells in the MEC.

## Introduction

The medial entorhinal cortex (MEC) is a six-layered cortical structure involved in episodic memory and spatial navigation. Layers I-III form the input and layers V-VI form the output domain to various cortical structures (Ramsden et al., 2015; Sürmeli et al., 2015; Witter et al., 2017). Projections to the hippocampus from the MEC primarily originate from the superficial layers, while outputs from the hippocampus terminate in the deep MEC layers (Canto et al., 2008). Through these reciprocal connections, circuits within the hippocampus and the MEC support spatial navigation and memory (Moser et al., 2008). The MEC consists of several types of spatially tuned cells (Hafting et al., 2005; Sargolini et al., 2006; Solstad et al., 2008) of which, the spatial firing patterns of “grid cells” are hypothesized to emerge locally. Grid cells fire in a repetitive hexagonal spatial pattern that spans the environment an animal navigates. An animal’s displacement, trajectory, or angular motion can be decoded from the discharge of grid cell populations (Moser et al., 2014). It is because of these properties that grid cells are also suggested to perform a function known as path integration (Mittelstaedt and Mittelstaedt, 1980; McNaughton et al., 2006).

Grid cells are particularly abundant in layer II of the MEC (Sargolini et al., 2006; Boccara et al., 2010), and *in vitro* experiments have shown that excitatory coupling in LII is present but sparse. Instead, the excitatory neurons are connected almost exclusively via strong lateral inhibition (Couey et al., 2013; Fuchs et al., 2016; Winterer et al., 2017). Consistent with this, *in vivo* optogenetic activation of parvalbumin (PV) expressing inhibitory neurons directly inhibits both grid cells and border cells, with grid cells providing a back projection onto PV cells (Buetfering et al., 2014). Finally, DREADD inactivation of the PV inhibitory cell populations, but not the somatostatin expressing inhibitory populations (SOM), disrupt the spatial firing pattern of grid cells (Miao et al., 2017). Computational attractor models have shown how excitation-inhibition-excitation (E-I-E) networks or recurrent inhibitory networks can generate grid cells (Bonnevie et al., 2013; Schmidt-Hieber and Nolan, 2017; Kang and Balasubramanian, 2019). Thus, several lines of evidence indicate that grid cells are under strong lateral inhibition from interneurons within the MEC. To this end, the role of excitatory and certain inhibitory cell populations has been studied extensively, but the interplay required to generate and maintain this Excitation/Inhibition (E/I) balance in the MEC remains elusive.

While studies have shown that interneurons within the MEC receive inhibitory projections from the medial septum (Gonzalez-Sulser et al., 2014; Fuchs et al., 2016), another form of disinhibition, conserved across cortical structures, is mediated by Vasoactive Intestinal Peptide (VIP) expressing interneurons. These cells are known to target both PV and SOM inhibitory cells as well as excitatory cells in various structures (Kepecs and Fishell, 2014; Batista-Brito et al., 2017; Kuchibhotla et al., 2017; Rhomberg et al., 2018; Krabbe et al., 2019; Garcia-Junco-Clemente et al., 2019). Thus, VIP interneurons are well-positioned to provide transient and selective breaks in the E/I balance by disinhibiting excitatory cells in other cortical regions (Letzkus et al., 2015; Guet-McCreight et al., 2020). The disinhibitory circuit motif has been well defined in the hippocampus (Tyan et al., 2014; Turi et al., 2019), auditory cortex (Pi et al., 2013), basolateral amygdala (Rhomberg et al., 2018; Krabbe et al., 2019), visual cortex (Pfeffer et al., 2013; Batista-Brito et al., 2017), and somatosensory cortex (Bayraktar et al., 2000; Lee et al., 2013; Prönneke et al., 2015). These studies show that VIP interneurons exhibit a range of molecular, electrophysiological, and morphological characteristics, all of which remain less well-defined in the MEC. Through this study, we aim to describe both anatomical and *in vitro* physiological properties of VIP cells in the MEC, with a focus on differences across both laminae and across the dorsal-ventral extent of the MEC. Our work thus adds to the growing body of studies that describe the anatomical characteristics and functions of various interneuron populations within the hippocampal-entorhinal circuits that support spatial navigation and memory.

## Materials and Methods

### Animals

All animal procedures were performed in accordance with the McGill University and Douglas Hospital Research Centre Animal Use and Care Committee (protocol #2015-7725). Animals were housed in a temperature-controlled room with a 12/12 h light/dark cycle and food and water ad libitum. Animals were housed with littermates. For this study, we crossed homozygous Vip-ires-cre (VIP^tm1(cre)Zjh^, The Jackson Laboratory, Bar Harbor, ME, USA) mice with homozygous Ai9 lox-stop-lox-tdTomato cre-reporter strain mice (RRID: IMSR_JAX:007905) to generate VIPcre/tdTom mice in which tdTom is exclusively expressed in cells that have cre recombinase. tdTom fluorescence was used to identify VIP cells. Using the three different approaches (molecular, electrophysiological, and morphological techniques) to characterize VIP cells in the MEC, we also test the specificity and efficacy of the cre driven mouse line.

### Immunohistochemistry (IHC)

VIPcre/tdTom mice, aged between 5–7 weeks were deeply anesthetized and intracardially perfused with 4% paraformaldehyde in PBS. Brains were dissected, post-fixed by immersion in the same fixative for at least 24 h before slicing. Free-floating sagittal sections (40 μm) of the entire MEC were cut using a vibratome (VT 1200S, Leica, Wetzlar, Germany). Every fourth section was collected for IHC. After slicing, the sections were washed in 1× PBS (3 × 5 min) and blocked with 1× PBS containing 0.45% fish gelatin and 0.25% Triton-X (3× 15 min each). Sections were then incubated with primary antibodies for 48 h at 4°C on a rotating shaker. The following primary antibodies were used: 1:500 rb-α-VIP Immunostar 722001/20077 (ImmunoStar, Dietzenbach, Germany), 1:500 Ms-α-PV PARV-19 monoclonal P3088 (Millipore Sigma, Saint Louis, Missouri, USA), 1:1,000 Ms-α-SOM (H-11) sc-74556 (Santa Cruz Biotechnology, CA, USA), 1:3,000 rb-α-Calretinin (CR) 7697 (Swant, Marly, Switzerland) and 1:2,000 rb-α-GABA A 2052 (Milipore Sigma). The sections were then subsequently rinsed in 1× PBS (3 × 5 min) and then incubated in Alexa Fluor 488-conjugated Ab raised against rabbit IgG and mouse IgG at 1:1,000 for 2 h at RT. After several washes with 0.3% PBS-T the sections were then mounted, and coverslipped with mounting medium Fluoromount-G and DAPI, 0100–20 (SouthernBiotech, Birmingham, AL, USA). For IHC against VIP and CR *n* = 3 animals and for staining against GABA *n* = 2 animals. For IHC against PV and SOM *n* = 1 animals.

### Image Acquisition and Data Analysis for Quantification

Quantification of VIP, CR, and GABA colocalization was performed using the optical fractionator method in StereoInvestigator software with a Zeiss ApoTome structured illumination device on a widefield microscope. Contours were drawn to delineate the superficial (LI-III) and deep layers (LIV-VI) of the MEC using DAPI as reference. While LIV is lamina dissecans in the MEC, we repeatedly found many tdTom cell bodies in between LIII (on the outer edge of the layer) and LV. Our observations are similar to those previously reported in Canto et al., 2008. Hence for all VIP cells located in deep layers, VIPtdTom somas found in this region (LIV) are included in the LIV-LVI category.

Using live counting, all markers were counted according to optical dissector inclusion-exclusion criteria at each cell’s widest point. Separate markers were used to count the number of tdTom (RFP), VIP/CR/GABA (GFP), and colocalized or double labeled cells (yellow).

### *In vitro* Electrophysiology

VIPcre/tdTom (*n* = 17 animals), of either sex, aged between 5–8 weeks were killed by decapitation and the brain was dissected and placed in an ice-cold high-sucrose solution in the following composition: 252 mm sucrose, 24 mm NaHCO_3_, 10 mm glucose, 3 mm KCl, 2 mm MgCl_2_, 1.25 mm NaH2PO_4_, and 1 mm CaCl_2_, continuously oxygenated with 95% O_2_/5% CO_2_; pH 7.3. Sagittal sections of 350 μm were cut using a vibratome, according to the method previously described by Pastoll et al (Pastoll et al., 2012). Following this, the sections were transferred to an artificial cerebrospinal fluid (aCSF) solution at RT (solution as above with 126 mm NaCl replacing sucrose, 2.5 mm KCl, and 2 mm CaCl_2_) for 30 min before recording. Slices were recorded in a bath aCSF heated to 35°C. VIPtdTom cells were identified by tdTom fluorescence using an upright BX51WI Olympus microscope with a 40x immersion objective (Olympus, Canada) and an X-cite Series 120Q fluorescence system (Lumen Dynamics). For whole-cell patch-clamp recordings, glass pipettes of 5–7 MΩ resistance were filled with an intracellular composition with the following components: 144 mm K-gluconate, 10 mm HEPES, 3 mm MgCl_2_, 2 mm Na2ATP, 0.3 mm GTP, and 0.2 mm EGTA; adjusted to pH 7.2 with KOH. Membrane potentials were not corrected for a junction potential of approximately −10 mV. VIPtdTom cells were recorded using a visually guided whole-cell patch-clamp technique, a Multiclamp 700B amplifier, a DigiData 1440A digitizer and pClamp10 software.

### Morphology of VIP Cells

For the morphology of VIP cells, cells were patched using the above intracellular solution with the addition of neurobiotin (0.4%, SP-1120–50, Vector Laboratories, ON, Canada). To obtain efficient biocytin fills, cells were subjected to a high amount of current at the end of each recording session (Jiang et al., 2015). Following this, the pipette was left undisturbed in whole-cell mode for 30 min to allow for the diffusion of neurobiotin through all neuronal processes. Finally, the pipette was retracted carefully to ensure that the cell membrane did not rupture, as this would result in incomplete fills or the loss of the soma. Slices were fixed in PFA (4% in PBS) at 4°C overnight. Fixed slices were incubated with streptavidin Alexa 488 (1:1,000, Jackson ImmunoResearch, West Grove, PA, United States) and NeuroTrace 647 (1:1,000) in 0.3% PBS-Triton overnight at room temperature. Slices were mounted on glass slides with Fluoromount G, 0100-01 (Southern Biotech). Biocytin-labeled VIP cells were imaged under the MBF Zeiss ApoTome microscope with a 20× magnification (Carl Zeiss, Germany). The cells were manually reconstructed using Neurolucida 11 software (MBF Bioscience, Williston, United States).

Immunostaining for CR following *in vitro* recordings in 350 μm sections: The protocol from Swietek et al., 2016 was adapted for immunostaining following a recording session. In brief, after recording, slices were fixed in PFA (4% in PBS) at 4°C overnight. The following day, they were rinsed in 0.3% PBS-T (3 × 15 min) before incubation with primary antibody, 1:2,000 rb-α-Calretinin (CR) 7697 (Swant, Marly, Switzerland) overnight at RT. The next day, the sections were rinsed in 0.3% PBS-T before incubating in streptavidin Alexa 647 (1:1,000) and Alexa Fluor 488-conjugated Ab raised against rabbit IgG (1:500) overnight at RT. After 3 × 10 min washes in PBS, the slices were mounted on glass slides and coverslipped with mounting medium Fluoromount-G and DAPI, 0100–20 (SouthernBiotech, Birmingham, AL, USA).

Contours between layers were marked using cells labeled with NeuroTrace or DAPI as a reference. Dendrites were distinguished from axons by the presence of spines. Initial axon segments were identified by their smooth appearance near the soma which later appeared discontinuous as the axon continued branching. Due to slicing techniques, neuronal processes may be cleaved, leaving abrupt endings visualized as ball-like structures at the end of the processes. The dendrites and axons of filled cells were noted for these abrupt endings and if present were excluded from the analysis. We also note that our reconstruction includes partial trees at least in the case of axons, that are not in continuity of the initial segment.

### Data Analysis and Statistics

Electrophysiological properties were analyzed with Clampfit10 Software. Recordings were kept for analysis if the spikes overshot 0 mV and access resistance was <30 MΩ. Membrane resistance (Rm) and access resistance (Ra) were measured in voltage clamp (vc) using pClamp10 software. The following properties were assessed in the current clamp (cc). Resting membrane potential (RMP) and spontaneous spiking were assessed over a 30 s recording with no holding current. I-V curves were generated from 20 pA current pulses ranging from −80 pA to +100 pA at the voltage sag-peak. To assess spike properties, cells were held at a holding potential (h.p.) of −65 mV and a series of 500 ms depolarizing current steps in 10 pA increment steps was applied. The step which elicited the first spike or action potential (AP) was used to assess rheobase, decay time, AP peak amplitude and AP half-width, and after-hyperpolarization (AHP) amplitude and time. The AHP time (ms) was measured from the time point the repolarization of the AP reached firing threshold to the maximum amplitude of the AHP.

Firing patterns generated on double the rheobase current were used to categorize the firing patterns based on the Petila convention (Ascoli et al., 2008) and were also used to analyze and determine peak interspike interval (ISI; for the first two spikes), steady-state ISI (ISI for the last two spikes). Spike frequency adaptation (SFA%) was determined using the following [(First Instantaneous Frequency − Last instantaneous frequency)/First Instantaneous Frequency]. Phase plots were analyzed in Clampfit10 Software for the first AP generated at double the rheobase current. To assess the hyperpolarization-activated sag current (I_h_), if present, a series of hyperpolarizing current steps was applied at a h.p. of −65 mV. The step which hyperpolarized the cell to −120 mV was used to calculate the sag amplitude, measured as the difference between peak and steady-state hyperpolarization. Procedures to measure the location of the cell along the dorsal-ventral axis and in a specific layer were modified from Pastoll et al. (2012). In brief, after each recording session, the pipette would be pulled out of the cell carefully from a whole-cell configuration to a cell-attached mode. Using an Olympus microscope with a 4× objective, images of the position of the pipette relative to the dorsal border of the MEC were taken and stitched together for each cell. This was performed to confirm the location of the recorded cell for a specific layer and its location along the dorsal-ventral axis of the MEC. A total of 60 VIP cells were patched and recorded.

To assess the morphological properties of the recorded cells, the reconstruction of 17 (LI-III = 11 cells; LIV-VI = 6 cells) neurons were quantified with Neurolucida 11 software (MBF Bioscience, USA). These neurons were considered for reconstruction as they had not visualized swellings in their axonal and dendritic arbors. Dendritic and axonal complexity were assessed using Sholl analysis in which concentric Sholl segments are generated starting from the center of the cell body (radial interval: 10 μm) and the number of process intersections per Sholl segment are recorded. Branched structure analysis was conducted to estimate the total length of the neural process contained within each layer of the MEC (Neurotrace/DAPI was used to distinguish between the different layers of the MEC). Data were not corrected for tissue shrinkage.

Statistical analysis was carried out using GraphPad prism software (San Diego, CA, USA). Data are presented as mean ± standard error of the mean (SEM) throughout the text and in Supplementary Tables 1, 2. Statistical significance was tested with two-tailed Mann-Whitney U test (layer differences, vertical and horizontal extent of neurite process) and linear regression (dorsal-ventral). R^2^ values are stated for the results of linear regression. A two-way analysis of variance (ANOVA) was performed to determine the length of neurite process present within in a layer. A *post hoc* Bonferroni test on all pairwise comparisons was performed to determine significance.

## Results

### Molecular Characterization of VIP Cells in the MEC

**Figure 1:**
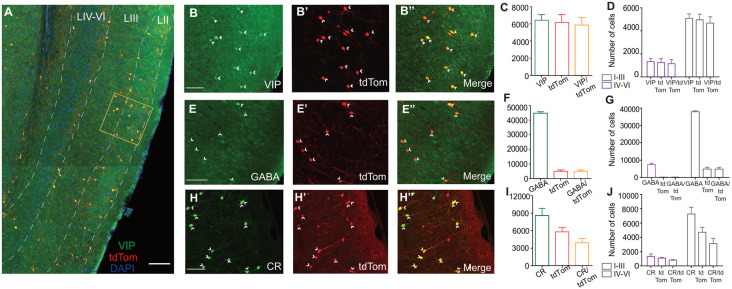
Specificity of cre-driven fluorescence for Vasoactive Intestinal Peptide (VIP) in VIPcre/tdTom mice and presence of colocalization with other molecular markers. **(A)** Co-expression of VIP (green) and tdtom (red) in a sagittal section of the MEC at low magnification 10x. Layers of the MEC (white dashed lines) marked using DAPI as reference. High magnification of framed area (orange box) is shown in **(B–B”)**. **(B–H”)** 20x maximum projection intensities of a 40-μm thick section of superficial MEC treated with IHC using αVIP AB **(B–B”)**, αGABA AB **(E–E”)**, and αCR AB **(H–H”)**. Fluorescence of cre-driven tdTomato expression is shown in red **(B’–H’)** and colocalization of the two channels is shown in **(B”–H”)**. The presence of colocalization is indicated by yellow cell bodies and white arrowheads. Graphs depicting the total number of cells present in the MEC **(C–I)** and distribution of somas in different layers of the MEC **(D–J)** for αVIP, αGABA , and αCR respectively are shown here. Percentages displayed for colocalization bar show the number of tdTom positive cell bodies which colocalize with the IHC marker. For IHCs performed for αVIP and αCR, *n* = 3 animals and for IHC performed against αGABA, *n* = 2 animals. Each bar represents a mean and error bars represent SEM. For panels **(B–H”)** scale bars are 250 μm. The scale bar in **(A)** is 500 μm.

We quantified the distribution of MEC VIP neurons in layer-specific and layer-independent manners by crossing transgenic VIP-ires-cre and tdTomato cre-reporter mice. To verify the validity of the mouse model and assess if the expression of tdTom in VIP cells is an effective way to identify VIP in a consistent manner, we performed IHC against VIP antibodies and other protein markers known to colocalize with VIP positive cells (Figure 1). We found that a total of 6,219.56 ± 607.81 cells expressed tdTomato (tdTom), of which 5,849.51 ± 633 cells were labeled with VIP IHC (Figure 1C). Thus, 94.05% of tdTom cells colocalized with VIP (VIPtdTom). When quantified by cortical layer, VIP cell bodies were not equally distributed. Superficial MEC (LI-III) had the highest proportion of VIPtdTom cell bodies (89.28%), while the deep MEC (LIV-VI) consisted of 19.71% of the cell bodies (4,673 ± 388.40 cells in the superficial layer vs. 1,153.43 ± 251.21 cells in the deep layer; Figure 1D). These results indicate that tdTom expression is highly specific (Figures 1A–D) for VIP-positive cells in the VIPcre/tdTom mouse line and thus suitable for the study. To ascertain if VIP cells express other molecular markers, we performed IHC against antibodies for GABAergic cells (Figures 1E,E′′), Calretinin (CR, Figures 1H,H′′) and estimated the percent of colocalization with tdTom. For Figures 1B–D and 1H–J, *n* = 3 animals, for Figures 1E–G, *n* = 2 animals.

We observed that all tdTom cells colocalized with GABA (4,945.75 ± 685.01 cells). Our results indicate that 11.05% of the total GABAergic population are positive for VIP (4,945.75 ± 685.01 tdTom cells of 44, 726.64 ± 727.24 GABAergic cells; Figure 1F), similar to previous reports in the cortex (Rudy et al., 2013; Prönneke et al., 2015; Tremblay et al., 2016). As seen in other structures, VIP cells expressed CR in the MEC. Of all the CR-expressing cells, 45.63% colocalized with tdTom (3,922 ± 521.05 CR-positive tdTom cells of 8,594.54 ± 682 CR cells) while 67.59% of tdTom cells colocalized with CR (3,922 ± 521.05 CRtdTom cells of 5,802 ± 520.72 tdTom cells; Figure 1I). This is in line with results of VIP cells in the hippocampus (Tyan et al., 2014) and basolateral amygdala (Rhomberg et al., 2018), but much higher than the 35% of colocalization observed in the visual cortex (Gonchar et al., 2008). We further looked at the distribution of CR-positive tdTom cells across the layers (Figure 1J). A large proportion (79.27%) of CR-positive tdTom cells were found in the superficial layers (3,109.44 ± 534.83 cells). Previous studies reported that VIP positive cells do not overlap with other molecularly distinct subpopulations such as PV and SOM (Prönneke et al., 2015). We observed a similar pattern in our experiments (Supplementary Figures 2A,B”).

### Electrophysiological Properties of VIP Neurons Vary Between Superficial and Deep Layers of the MEC

**Figure 2:**
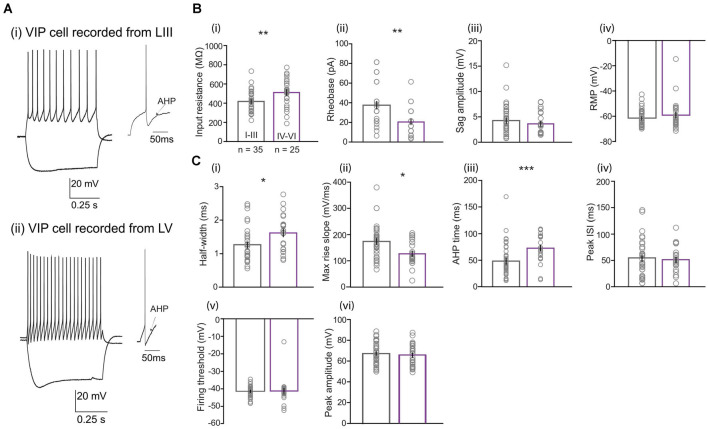
Comparison of electrophysiological properties between LI-LIII and LIV-LVI VIP cells. **(A)** Examples of membrane potential responses to current steps (−80 pA for lower trace and +100 pA for upper trace) recorded from VIP cells located in the (i) superficial (LIII) and (ii) deep (LV) layers of the MEC. Examples of afterhyperpolarizations (AHP) following action potentials are also shown for the respective cells. **(B)** Passive and **(C)** active membrane properties for 60 VIP cells separated into layer I–III (*n* = 35) and layers IV–VI (*n* = 25). Except for (iii) sag amplitude where layer I–III (*n* = 33) and layers IV–VI (*n* = 24) and (iv) peak ISI where layer I–III (*n* = 33) and layers IV–VI (*n* = 22). Each bar represents a mean and error bars represent SEM. Significant differences were found in **(B)** (i) input resistance, (ii) rheobase and **(C)** (i) half-width, (ii) max rise slope, and (iii) AHP time (*p < 0.05, **p < 0.01, ***p < 0.0001).

Next, we determined the electrophysiological properties of 60 VIP cells from all layers of the MEC (LI-III *n* = 35; LIV-VI *n* = 25; Figure 2A). Two passive membrane properties were significantly different between the two populations (Figure 2B): VIP neurons in LIV-VI had higher input resistance [IR; Figure Figure 2B(i)] compared to neurons present in LI-III (511.51 ± 28.91 vs. 418 ± 18.63 MΩ, Mann Whitney *U* = 257.0, *p* = 0.0070). Consistent with this difference, the amount of current [rheobase; Figure Figure 2B(ii)] required to generate an action potential (AP) in deep MEC was lesser than the superficial VIP cells (20.22 ± 2.66 vs. 36.85 ± 3.64 pA, Mann Whitney *U* = 222.5, *p* = 0.0012). Layer differences were also observed for active membrane properties (Figure 2C): Cells in the superficial layers had a smaller AP half-width compared to VIP cells in the deep layers [1.25 ± 0.008 vs. 1.6 ± 0.103 ms, Mann Whitney *U* = 260, *p* = 0.008; Figure Figure 2C(i)], accordingly significant difference was observed in the maximum rise slope of spikes [Figure Figure 2C(ii)] between superficial and deep VIP cells (174.30 ± 10.69 vs. 126.75 ± 8.85 mV/ms, Mann Whitney *U* = 215, *p* = 0.010). We also found deep VIP cells to have a much longer duration in AHP time compared to superficial cells [72.78 ± 5.21 vs. 56.78 ± 9.51 ms, Mann Whitney *U* = 207, *p* = 0.0006; Figure Figure 2C(iii)]. No significant differences were observed in properties such as resting membrane potential (population average of −60 ± 1.17 mV), voltage sag amplitude (4.05 ± 0.35 mV), firing threshold (−41.28 ± 0.77 mV), peak amplitude (6.62 ± 1.3 mV), half-amplitude (33.22 ± 0.64 mV), AHP amplitude (−11.10 ± 0.67 mV), and steady-state inter-spike interval (37.59 ± 3.65 ms; Supplementary Table 1). I-V curves and phase plots for VIP cells located in superficial and deep cells are included in Supplementary Figure 2.

### Electrophysiological Properties of VIP Cells Vary Along the Dorsal-Ventral (D-V) Axis of the MEC

**Figure 3:**
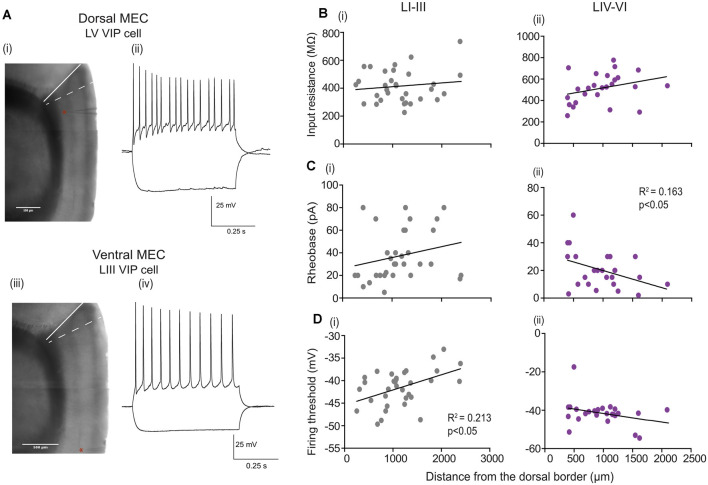
Passive and active properties as a function of layer and location along the Dorsal-Ventral axis. **(A)** (i and iii) Parasagittal brain slice under the experimental microscope (aligned and blended composites of low magnification 4× images). The border between the postrhinal cortex and sensory cortex is indicated by a white solid line, while the dorsal border of the MEC is indicated by a white dashed line. The position of the dorsal cell in **(A)** Located 286 mm from the dorsal MEC border, and the ventral cell shown in **(A)** (iv) located 2,128 μm from the dorsal MEC border, are indicated by a red star near the recording pipette in the respective sections. **(A)** (ii and iv) Examples of membrane potential responses to −80 pA (lower traces) and +80 pA (upper traces) current step recorded from (ii) LV VIP cell and (iv) LIII VIP cell. **(B–D)** Passive and active properties plotted as a function of location and layer for individual VIP cells recorded from the superficial layers LI–III: 35 cells **(B–D)** (i) and **(B–D)** (ii) deep layers LIV–VI: 25 cells. In all panels, black lines indicate linear fits to the data. For cells located in the deep MEC significance in slope for **(C)** (ii) rheobase is observed, and for cells located in the superficial layers significance in slope for **(D)** (i) firing threshold is observed.

Grid cells in the MEC show variation in their grid scales along the D-V axis (Hafting et al., 2005; Sargolini et al., 2006; Brun et al., 2008). To understand the potential mechanism underlying this topographical organization, previous work has investigated if the active and passive membrane properties of the different classes of excitatory cells differed depending on their location from the dorsal border. Prior studies have shown that stellate cells exhibit a gradient in the input resistance (IR), membrane time constant and rheobase (Garden et al., 2008; Giocomo and Hasselmo, 2008), and hyperpolarization-activated cation current (I_h_; Giocomo et al., 2011). We asked if VIP cells show a similar gradient in their properties along the D-V axis (Figures 3A–D). We recorded 58 VIP-expressing cells (LI-III = 33; LIV-VI = 25 cells) along the D-V axis (we excluded two cells due to an inability to quantify the position of the recording pipette). Since we observed layer differences in the electrophysiological properties, we further asked if passive and active properties varied as a function of the cell’s laminar position. We found that for VIP cells located in deep MEC, the rheobase [Figure Figure 3C(ii)] was much larger for neurons located close to the dorsal border as compared to those recorded at more ventral locations (slope = −0.012 ± 0.005 pA *R*^2^ = 0.163, *p* = 0.045). Interestingly, the input resistance [Figure Figure 3B(ii)] and the firing threshold of these cells showed no dependence on the location of the cell along the D–V axis [Figure Figure 3D(ii)]. For cells located in the superficial layers, the firing threshold [Figure Figure 3D(i)] steeply increased with the cell’s distance from the dorsal border (slope = 0.0,033 ± 0.0,011 mV *R*^2^ = 0.213, *p* = 0.006), while other properties showed no dependency on the location of the cell along the dorsal border (data not shown).

### VIP Cells Show Heterogeneity in Their Firing Patterns

**Figure 4:**
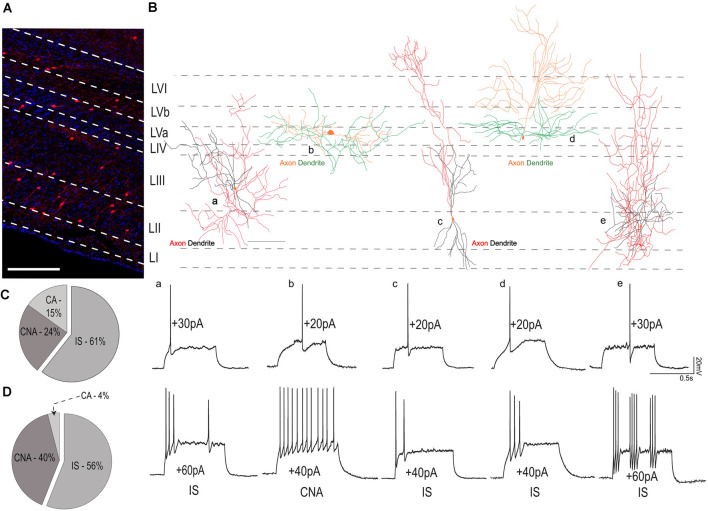
Variability in firing patterns of VIP cells from layers I-III and IV-VI. **(A)** Distribution of VIP cells (tdTom) in the MEC, the layers have been marked using DAPI as reference. The scale bar is 500 μm. **(B)** The reconstructed morphology of five individual VIP cells is shown in a schematic of the MEC. For all cells, somas are colored in orange. For cells recorded in superficial MEC axonal and dendritic processes are colored in red and black, respectively. For cells recorded from layers IV-VI, axons are in orange while dendrites are colored green. Below, electrophysiological recordings from the respective cells at rheobase (upper trace) and action potential firing pattern at suprathreshold current (lower traces). The scale bar in **(B)** is 100 μm. Percentages of various firing patterns observed in the **(C)** superficial layers of the MEC and **(D)** deep layers of the MEC. Abbreviations name the firing pattern after the Petila convention (Ascoli et al., 2008; IS, Irregular spiking; CA, Continuous adapting; CNA, continuous non-adapting).

Next, we sought to investigate the firing patterns of VIP neurons in the MEC. As reported in previous studies, VIP cells were never fast-spiking, but showed the following firing patterns, according to the description in the Petila convention (Ascoli et al., 2008): irregular spiking (IS, 60%), continuous non-adapting (CNA, 30%), and continuous adapting (CA, 10%; Supplementary Figure 1C). We further investigated the distribution of these firing patterns within the MEC layers. Distribution of VIP cells across the different layers of the MEC can be seen in Figure 4A. In the superficial layers (*n* = 35 cells), 61% of the cells had IS firing pattern, while CNA and CA comprised the remaining 24% and 10% respectively (Figure 4C). In the deep layers (*n* = 25 cells), 56% of the cells had IS firing pattern while 40% were CNA and the remaining 4% were CA (Figure 4D).

### Morphological Features of Individual VIP Neurons

**Figure 5:**
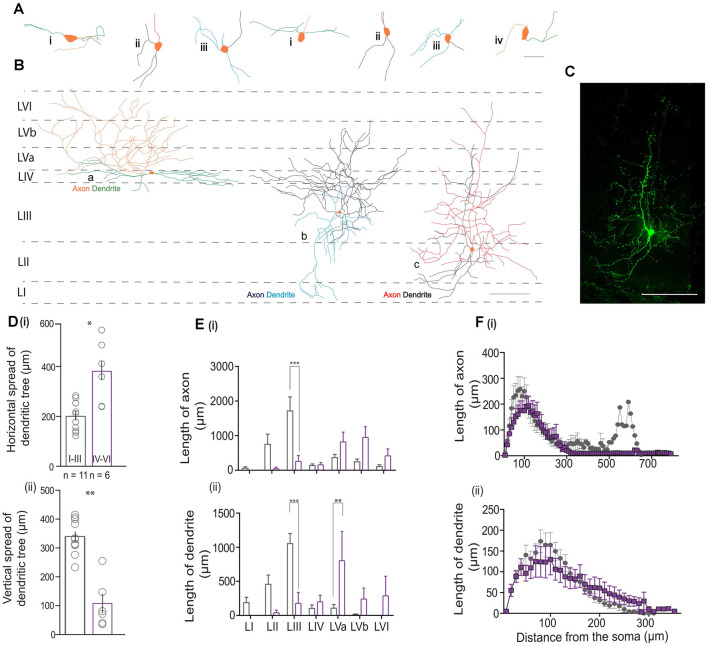
Morphometric analysis of VIP cells. **(A)** Magnified views of the somatodendritic and axonal processes representing the following morphologies: i—horizontal bitufted, ii—vertical bitufted, iii—multipolar, and iv—unipolar architectures. The scale bar is 50 μm. Please note that in **(A)** (ii—right), the axon originates from a primary dendrite. **(B)** Schematic of the MEC with neurolucida reconstructions depicting the three predominant morphologies observed in the data; horizontal bitufted in L4 (a; axons in orange, dendrites in green), multipolar VIP neuron in LIII (b; axons in dark blue and dendrites in light blue), and vertical bitufted morphology seen in L2 (c; axons in red, dendrites in blue). Scale bars are 100 μm. For all cells, the soma is colored in orange. **(C)** Maximum projection intensity of 207 z-stacks of 0.5 μm depth taken in 20x magnification of reconstruction seen in **(B)** (c) of the MEC filled with neurobiotin (GFP). Scale bar is 100 μm. **(D)** Plots depicting the (i) vertical and (ii) horizontal spread of the dendritic process. Each bar represents a mean and SEM. **(E)** Graphs assessing the length of processes present in a specific layer for (i) axons and (ii) dendrites. Each bar represents a mean and SEM. **(F)** Morphological features of (i) axons and (ii) dendritic arbors obtained from Sholl analysis. Each bar represents a mean and SEM. For all graphs, LI–III = 11 cells, LIV–VI = 6 cells: ***p < 0.001, **p < 0.01, *p < 0.05.

To study the morphological features of VIP cells, we reconstructed and analyzed the dendritic and axonal morphology of 17 VIP neurons from all layers of the MEC. We found that most VIP cells presented with a bitufted [Figures Figures 5A(i and ii) and Figure 5C] or a multipolar morphology [Figure Figure 5A(iii)]. Our reconstructions indicate that deep VIP cells exhibited dense dendritic arborization. Indeed, we found that cells in the deep layers have a greater horizontal spread of their dendritic trees [Figure Figure 5D(i); 385.01 ± 46.9 vs. 202.70 ± 16.98 μm, Mann Whitney *U* = 8.00, *p* = 0.013], while the dendrites of cells in LI-III are spread across the vertical column [Figure Figure 5D(ii); 339 ± 16.60 vs. 107 ± 31.15 μm, Mann Whitney *U* = 1.00, *p* = 0.001]. We found no significant differences in the horizontal or vertical extent of the axonal arbors between the two groups (data not shown). Given the difference in the dendritic expanse, we asked if the location of the cell influenced its projection to different layers. Cells in the superficial layers extended their axons well into the deep layers, however, deep VIP cells made almost no contact with the superficial layers [Figure Figure 5E(i)]. Layer III had extensive dendritic arborizations from superficial cells (*p* < 0.001, *t* = 4.487), while dendrites from the deep layers rarely extended beyond layer IV and thus their projections were more local [*p* < 0.01, *t* = 3.573; Figure Figure 5E(ii)]. A Sholl analysis revealed that most dendrites and axons were found locally near the soma (≤300 μm) for both groups (Figure 5F). However, it appears that the dendritic spread [Figure Figure 5F(ii)] for cells in the deep layers is much broader, while for superficial cells, it peaked at 100 μm from the soma.

## Discussion

Using *in vitro* whole-cell patch-clamp recordings, we investigate the distribution, morphology, active and passive membrane properties of VIP interneurons in the MEC. We validate that our VIP cre-driver mouse line is efficient and a suitable model system for studying the VIP population in the MEC. Our results confirm that VIP neurons in the MEC are a heterogenous population of cells, similar to reports from the hippocampus (Tyan et al., 2014), barrel cortex (Prönneke et al., 2015), and the BLA (Rhomberg et al., 2018). This study also extends previous findings on VIP cells in the MEC (Ferrante et al., 2017) in the following ways: we show evidence for a gradient in both the active and passive membrane properties of VIP cells along the D-V extent of the MEC, and that these differences are preserved between VIP cells in superficial and deep layers. Our results further indicate that VIP cells present with three different firing patterns, of which, irregular spiking cells are the most prominent type. Furthermore, morphological differences between superficial and deep VIP cells indicate that the input and output domains for superficial VIP cells are different from those seen in VIP cells located in the deep layers.

### VIP Cells Show Heterogeneity in Their Electrophysiological and Morphological Properties

In our dataset, we find that the heterogeneity in firing patterns in VIP cells are similar to previous reports from other cortical areas. However, the predominance of IS firing patterns in our dataset differs from some of these previous studies. This increase in IS firing pattern could be explained by the number of VIP cells that co-express CR in the MEC. Previous studies from the hippocampus (Tyan et al., 2014) and other cortical structures (Guet-McCreight et al., 2020) report an increase in the incidence of IS firing patterns when the cell co-expresses both VIP and CR. In addition to this, previous studies have also noted that VIP cells that colocalize with CR are disinhibitory in nature (Tyan et al., 2014; Turi et al., 2019; Guet-McCreight et al., 2020). From our IHC experiments, we find that 67.59% of the total tdTom cells colocalize with CR. Interestingly, we observe that 60% of the total VIP population in the MEC exhibited IS firing patterns. Taking layers into consideration, we see that 79.27% are positive for both CR and VIP markers. This could be reflected by the 61% incidence of IS firing patterns we observe in the superficial layers. However, it is also possible that VIP positive cells that co-express CR do not exhibit IS firing patterns as shown in Goff and Goldberg (2019). To ascertain if this is the case in the MEC, in a subset of our recorded and biocytin-filled cells, we labeled for CR and noted if the presence or absence of this calcium-binding protein made any distinction among the VIP positive cells. Of the four cells tested, two were found to be positive for CR (Supplementary Figures 3A–C′′′) and the presence of CR made no distinction among VIP cells with different firing patterns (two out of four cells that expressed CR had IS and CNA firing pattern (Supplementary Figures 3D–H), while the other two cells also showed the same firing patterns). We note that the sample size from this experiment is low and further work is required to determine if the observations hold true for a larger population of VIP cells in the MEC. In addition to this, in our recordings, we do not find VIP cells with a bursting firing pattern, a characteristic of VIP cells previously reported (Prönneke et al., 2020). Some studies attribute that cells that exhibit this firing characteristic belong to the CCK family (He et al., 2016). The presence of high numbers of CR positive tdTom cells as seen in our IHC experiments may explain this discrepancy. While this predominance may indicate a primarily disinhibitory circuit motif in the MEC, it remains to be established if the post-synaptic targets of VIP cells in the MEC are interneurons or if excitatory cells are also targeted by this population of cells. In terms of other electrophysiological properties, our population averages for superficial VIP cells are similar to a prior report, with the exception of a higher percentage of VIP cells with IS firing patterns (Ferrante et al., 2017).

The position of VIP cells within the MEC is associated with different morphological and electrophysiological properties. In terms of morphological features, we noticed both superficial and deep VIP cells present with similar dendritic and axonal architecture. However, their location in a specific layer dictates their projection patterns. Cells in the superficial layers had either a multipolar or vertical bitufted morphology enabling them to span the entire cortical column, similar to the observations from the barrel cortex (Bayraktar et al., 2000; Prönneke et al., 2015). These cells are well-positioned to receive and transmit incoming information to the entire MEC cortical mantle, while the cells in the deep MEC are found to exclusively cater to the deeper layers of the MEC. Furthermore, we notice that as the cells are located increasingly outwards from the MEC (from LI to LVI), the tendency of the axonal fibers to innervate the superficial layers decreases. Interestingly, this similarity in the neurite processes between superficial and deep MEC is also observed in VIP cells in the barrel cortex (Prönneke et al., 2015). We also note that in some VIP cells [Figures Figures 4B(a), 5B(b)], the dendritic and axonal morphologies are similar to those observed in the BLA (Rhomberg et al., 2018), an unlaminated structure. In the BLA, VIP cells are known to target both PV, SOM interneurons, and to a lesser extent pyramidal cells (Krabbe et al., 2019). It remains to be seen if similar connectivity is observed in the MEC.

We also identified differences in several electrophysiological properties between layers, notably in input resistance and rheobase. As a population, MEC VIP cells, are highly excitable, much like their counterparts in other structures (Kepecs and Fishell, 2014; Tyan et al., 2014; Ohara et al., 2018; Rhomberg et al., 2018; Guet-McCreight et al., 2020). From the current dataset, we found that deep VIP cells are more excitable than superficial cells. Moreover, we saw a gradient in this intrinsic excitability wherein cells located in ventral MEC require less current to fire an AP compared to cells in dorsal MEC. The gradients in excitability that we report could provide a gating mechanism that prioritizes certain outputs from the MEC as deep MEC sends projections to various intrathalamic structures and LII of the MEC (Sürmeli et al., 2015; Ohara et al., 2018) whereas superficial MEC primarily projects to the hippocampus (Canto et al., 2008).

## Functional Implications

There is a growing body of work suggesting that VIP interneurons are critical for facilitating associative learning and maintenance of the E/I balance in various cortical structures. In most structures, VIP cells located in layers I-III, the input layers of the cortex, innervate SOM, PV cells (Pi et al., 2013; Krabbe et al., 2018; Guet-McCreight et al., 2020) and thus provide for a break from ongoing inhibition in excitatory cells in the superficial layers. In the MEC, the majority of the VIP population (89%) is found in LI-III which forms its primary input domain (Witter et al., 2017). Furthermore, morphologies of VIP cells from LI-III show that these cells are well-positioned to transmit incoming information to cells within their layer and across the different layers of the MEC, thus making them likely candidates to target other interneurons and excitatory cells across the MEC. Our findings also show that a minority of VIP cells may not send projections to the superficial layers. Their specific functional role within the MEC circuit will require further studies. Through our experiments, we show that the VIPcre/tdTom mice line are suitable models for future experiments to determine the functions of this cell population *in vivo*.

## Data Availability Statement

The raw data supporting the conclusions of this article will be made available by the authors, without undue reservation.

## Ethics Statement

The animal study was reviewed and approved by McGill University and Douglas Hospital Research Centre Animal Use and Care Committee.

## Author Contributions

All authors helped to conceive and plan this study. SB collected and analyzed all data. SB and MB wrote the manuscript. All authors contributed to the article and approved the submitted version.

## Conflict of Interest

The authors declare that the research was conducted in the absence of any commercial or financial relationships that could be construed as a potential conflict of interest.

## Publisher’s Note

All claims expressed in this article are solely those of the authors and do not necessarily represent those of their affiliated organizations, or those of the publisher, the editors and the reviewers. Any product that may be evaluated in this article, or claim that may be made by its manufacturer, is not guaranteed or endorsed by the publisher.
