# Supplementary material for: A Characterization of the Electrophysiological and Morphological Properties of Vasoactive Intestinal Peptide (VIP) Interneurons in the Medial Entorhinal Cortex (MEC)
Source: Front Neural Circuits. 2021 Jul 23;15:653116. doi: 10.3389/fncir.2021.653116 (PMC8378838; doi:10.3389/fncir.2021.653116)
Supplement: Supplementary file 1 [file Data_Sheet_1.pdf]

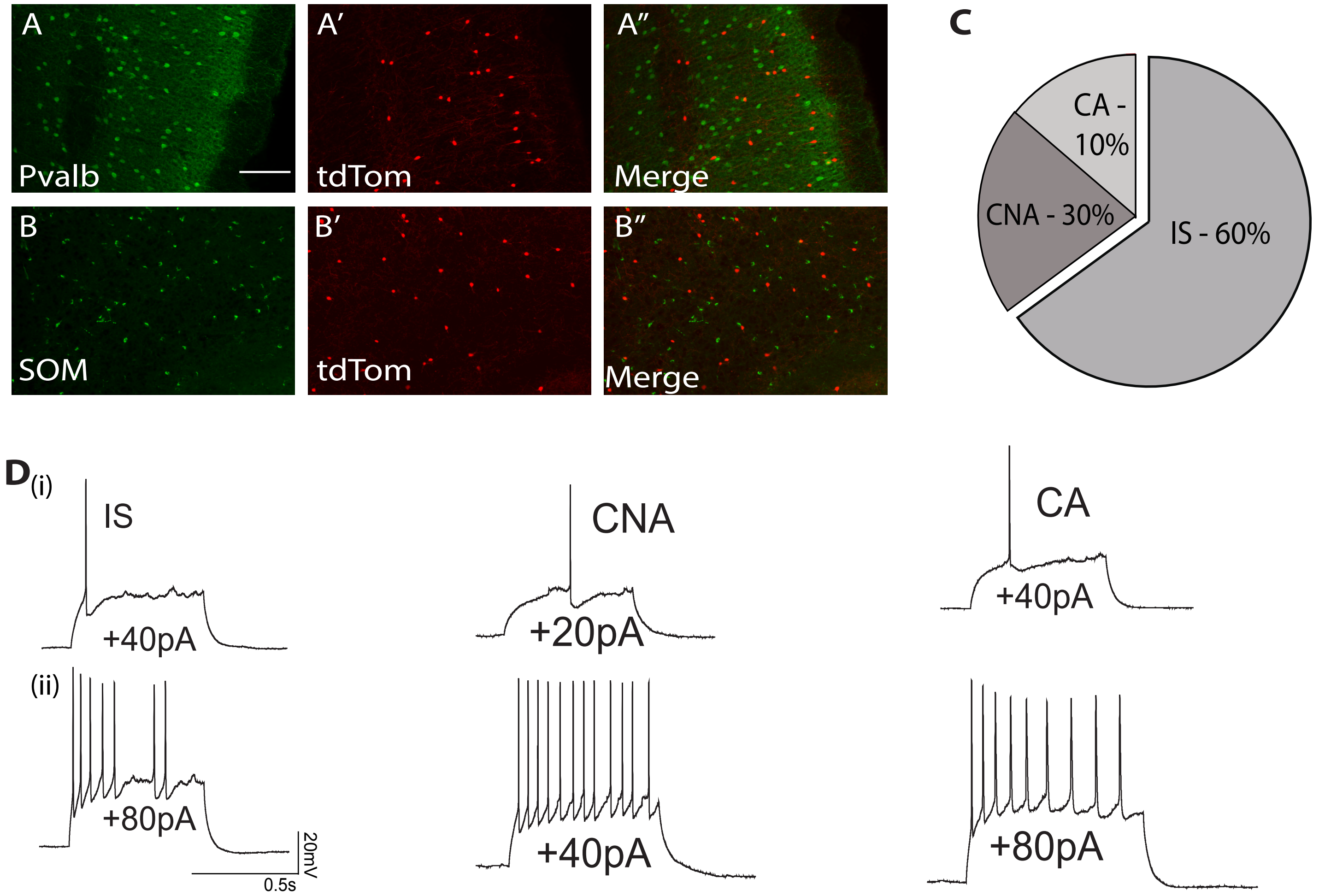

Supplementary Figure 1

(A-B”) Maximum projection intensity of a 40-µm thick sagittal section of superficial MEC treated with IHC using αPV AB (A-JA”) and αSst AB (B-B”). Scale bar is 250µm

(C) Percentage of action potential firing patterns observed in VIP cells in the MEC

(D) Examples of membrane potential responses to current steps recorded at (i) rheobase and (ii) suprathreshold current. Abbreviations in C and D name the firing pattern after the Petila convention (Ascoli et al.2008; IS, Irregular spiking; CA, Continuous adapting; CNA, continuous non-adapting).

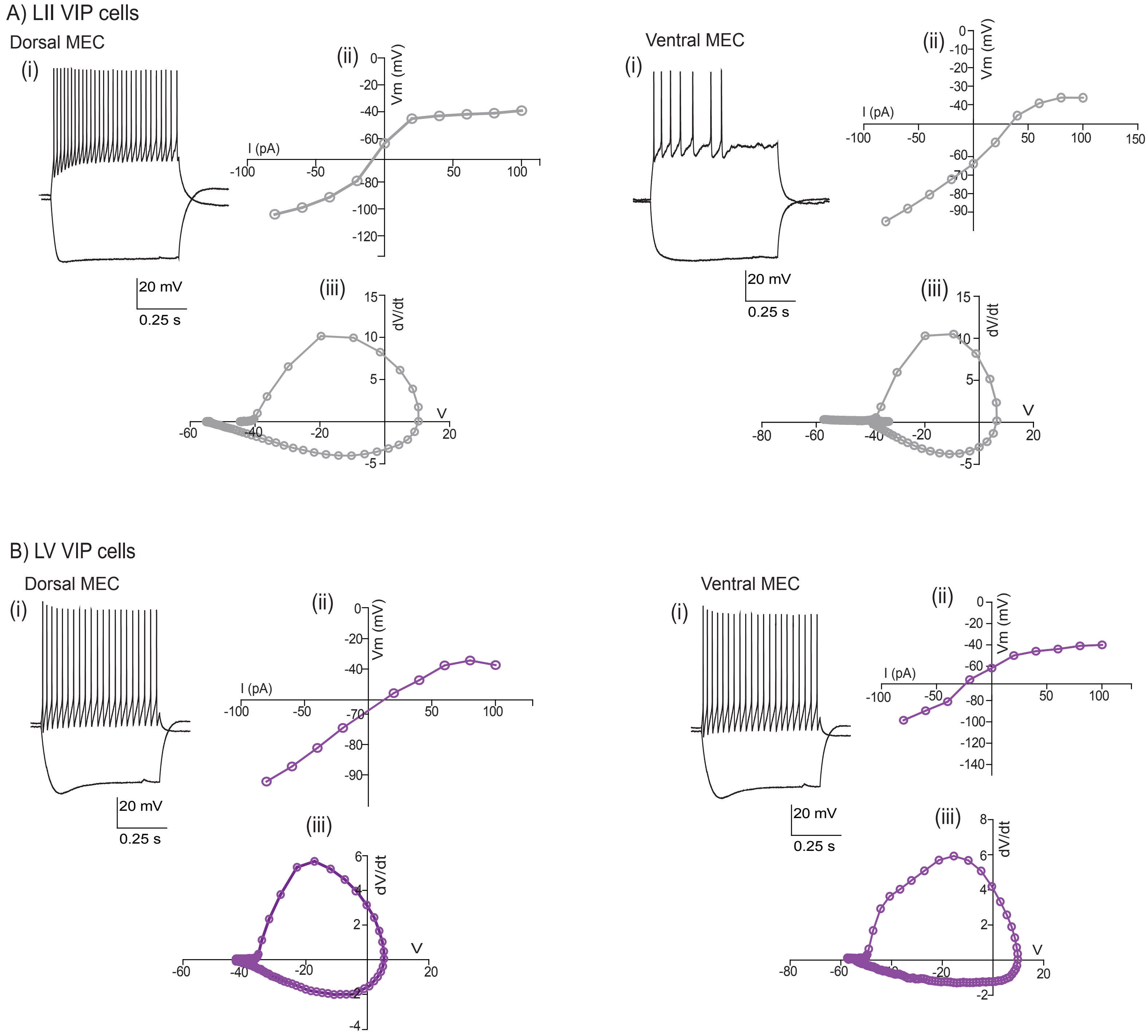

Supplementary Figure 2

VIP cells located in the A) superficial (LII) and (B) deep (LV) layers of the MEC along the dorsal and ventral axis. In all panels, (i) Examples of membrane potential responses to current steps (-80pA for lower trace and +100pA for upper trace), (ii) Current-voltage response (I-V curve) for each voltage response to varying current step at voltage sag-peak and (iii) Phase plot of first AP to current step double the rheobase current.

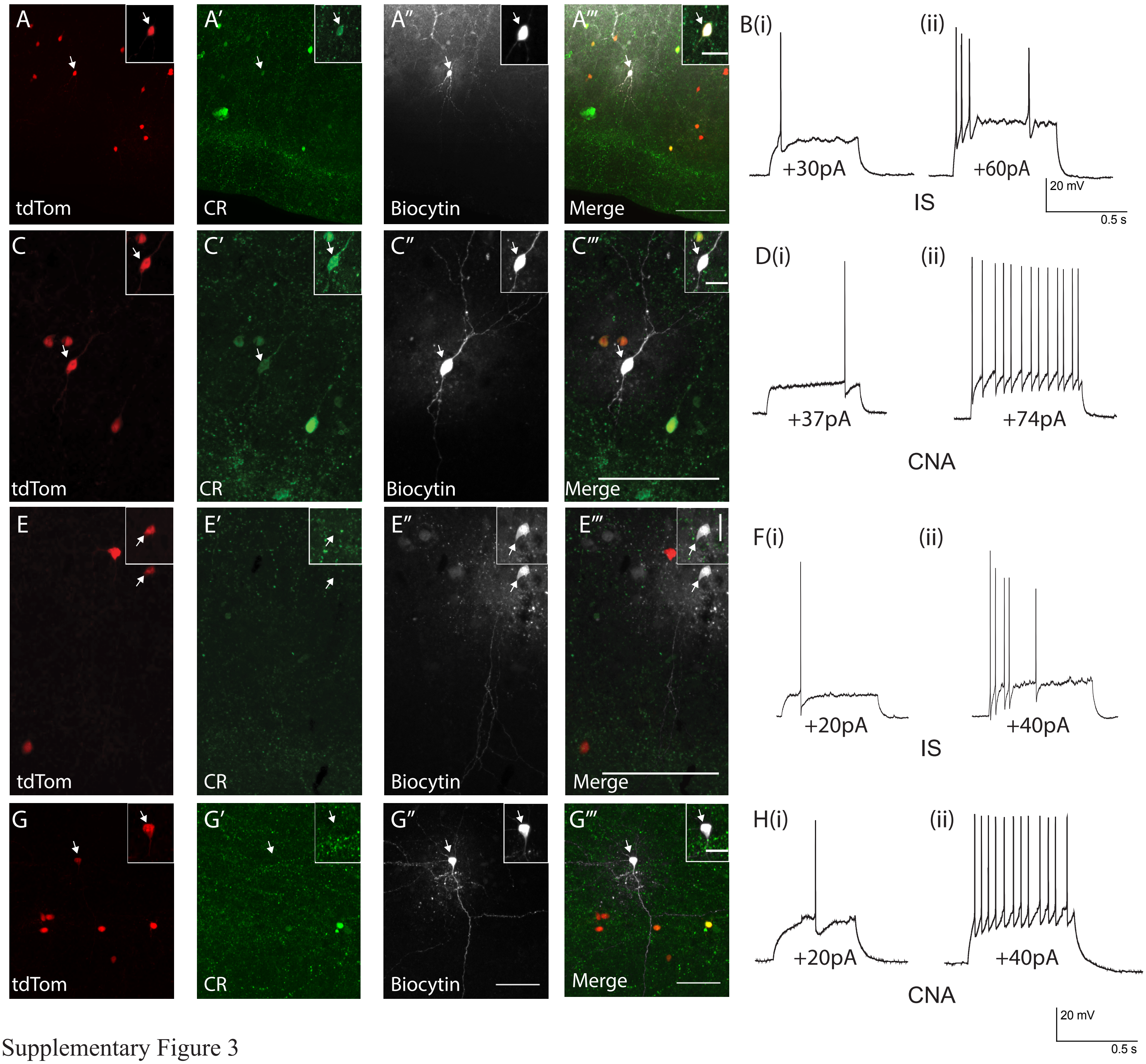

Supplementary Figure 3
Examples of VIP cells that show or lack immunoreactivity for Calretinin (CR).
Parasagittal sections of the MEC were processed for immunoreactivity for CR after an in vitro recording session. (A-E’’’) White arrow heads mark the presence of VIP (tdTom), CR (GFP) and Biocytin (gray) in their respective channels, in A’’’ and E’’’ colocalization of all three channels indicates presence of CR in the recorded cell.
In panels B and F are shown the electrophysiological recordings from the respective cells at (i) rheobase and action potential firing pattern at (ii) suprathreshold current.
In panels E and G the absence of CR marker in the recorded cell is visualized in E’ and G’.
The morphology of the recorded cell in G is multipolar. In all other panels, the cells display a bitufted morphology. Scale bars in insets are 20µm. Scale bar in G’’’ is 60µm. In all other panels the scale bar is 100µm. Abbreviations name the firing pattern after the Petila convention (Ascoli et al.2008; IS, Irregular spiking; CA, Continuous adapting; CNA, continuous non-adapting)

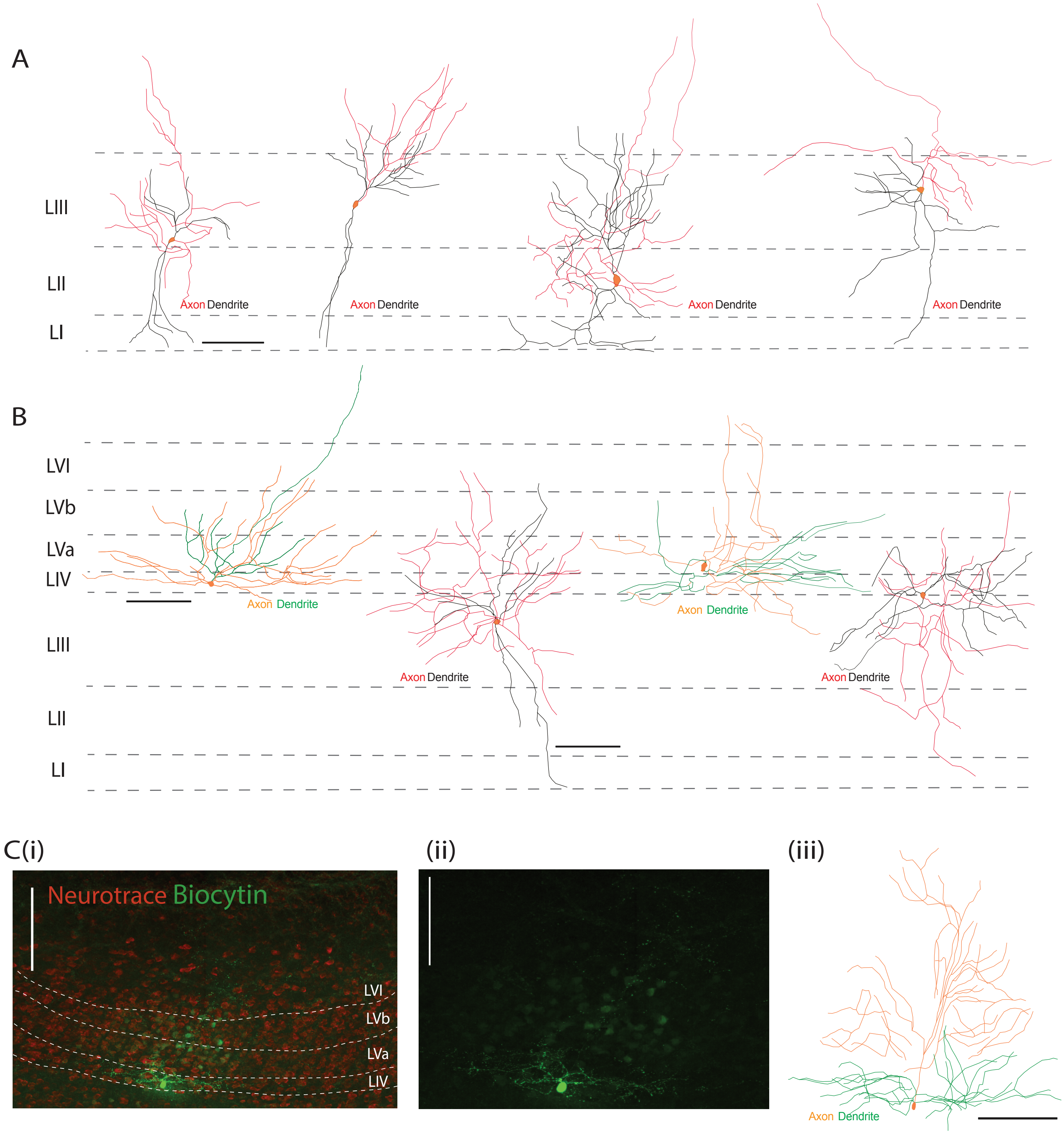

Supplementary Figure 4

(A) The reconstructed morphology of 4 individual VIP cellsrecorded from the superficial layers is shown in a schematic of the MEC. For all cells soma are coloured in orange and axonal and dendritic processes are coloured in red and black respectively. Scale bar is 100µm.

(B) The reconstructed morphology of 4 individual VIP cells is shown in a schematic of the MEC. For all cells soma are coloured in orange. For cells recorded in superficial MEC axonal and dendritic processes are coloured in red and black respectively. For cells recorded from layers IV-VI, axons are in orange while dendrites are coloured green. Scale bar is 100µm.

(C) Maximum projection intensity of a VIP cell filled with neurobiotin recorded from the deep layers of the MEC. (C(i))The layers of the MEC in C(i) are makred with neurotrace as reference. Reconstruction of recorded cell is seen in C(iii). Soma is coloured in orange and axons and dendrites are coloured in orange and green respectively.

Scale bar in C(i & ii) is 150µm and scale bar in C(iii) is 100µm.

Table 1

Properties measured	LI-III (n = 35)	LIV-VI (n = 25)	t test
Input resistance (MΩ)	418 ± 18.63	511.51 ± 28.9	p = 0.007
Rheobase (pA)	36.85 ± 3.64	20.22 ± 2.66	p = 0.001
Sag amplitude (mV)	4.17 ± 0.51 (n = 33)	3.55 ± 0.43 (n = 24)	p = 0.605
Decay tau (ms)	9.93 ± 2.45	14.17 ± 3.95	p = 0.264
RMP (mV)	-61.14 ± 1.12	-41.16 ± 1.62	p = 0.401
Half-width (ms)	1.25 ± 0.08	1.6 ± 0.103	p = 0.008
AHP time (ms)	56.78 ± 9.51	72.78 ± 5.21	p = 0.0006
Max rise slope (mV/ms)	174.30 ± 10.69	126.75 ± 8.85	p = 0.010
AHP amplitude (mV)	-11.68 ± 1.12	-11.16 ± 0.89	p = 0.928
Peak amplitude (mV)	67.28 ± 1.71	65.71 ± 2.09	p = 0.594
Half-amplitude (mV)	33.64 ± 0.85	32.65 ± 0.98	p = 0.533
Peak ISI (ms)	49.48 ± 6.26 (n = 33)	45.01 ± 5.8 (n = 22)	p = 0.870
Steady state ISI (ms)	34.36 ± 4.29 (n = 32)	29.94 ± 6.06 (n = 20)	p = 0.318
Spike frequency adaptation %	-9.12 ± 23.7 (n = 32)	32.09 ± 8.50 (n = 20)	p = 0.958
Firing threshold	-41.36 ± 0.65	-41.16 ± 1.62	p = 0.5739

Table 1. Tabulation of all passive and active membrane properties measured for VIP cells located in superficial (LI-LIII) and deep (LIV-LVI) layers in the MEC. p values obtained from t-test.

Table 2

Properties measured	Population average (n = 60)
Input resistance (MΩ)	457.01 ± 17.2
Rheobase (pA)	29.92 ± 2.62
Sag amplitude (mV)	4.05 ± 0.35 (n = 57)
Decay tau (ms)	11.69 ± 2.19
RMP (mV)	-60 ± 1.17
Half-width (ms)	1.40 ± 0.06
AHP time (ms)	63.45 ± 6.05
Max rise slope (mV/ms)	154.49 ± 7.85
AHP amplitude (mV)	-11.76 ± 0.67
Peak amplitude (mV)	66.62 ± 1.33
Half-amplitude (mV)	33.22 ± 0.64
Peak ISI (ms)	52.07 ± 4.35 (n = 55)
Steady state ISI (ms)	37.59 ± 3.65 (n = 52)
Spike frequency adaptation %	2.62 ± 14.45 (n = 52)
Firing threshold (mV)	-41.280 ± 0.77

Table 2. Tabulation of all passive and active membrane properties measured for all VIP cells located across all layers.
